# Feather Waste Biodegradation and Biostimulant Potential of *Gordonia alkanivorans* S7: A Novel Keratinolytic Actinobacterium for Sustainable Waste Valorization

**DOI:** 10.3390/ijms26136494

**Published:** 2025-07-05

**Authors:** Katarzyna Struszczyk-Świta, Piotr Drożdżyński, Paweł Marcinkowski, Aleksandra Nadziejko, Magdalena Rodziewicz, Bartłomiej Januszewicz, Magdalena Gierszewska, Olga Marchut-Mikołajczyk

**Affiliations:** 1 Institute of Molecular and Industrial Biotechnology, Faculty of Biotechnology and Food Sciences, Lodz University of Technology, 2/22 Stefanowskiego Street, 90-537 Lodz, Poland; piotr.drozdzynski@p.lodz.pl (P.D.); olga.marchut-mikolajczyk@p.lodz.pl (O.M.-M.); 2 Institute of Materials Science and Engineering, Faculty of Mechanical Engineering, Lodz University of Technology, 1/15 Stefanowskiego Street, 90-537 Lodz, Poland; bartlomiej.januszewicz@p.lodz.pl; 3 Department of Physical Chemistry and Polymer Physical Chemistry, Faculty of Chemistry, Nicolaus Copernicus University in Toruń, 7 Gagarina Street, 87-100 Toruń, Poland; mgd@umk.pl

**Keywords:** keratinous waste degradation, *Gordonia*, actinomycetes, keratin hydrolysate, waste-derived bioproducts

## Abstract

The poultry industry produces significant quantities of keratin-rich waste, primarily feathers, whose traditional disposal methods—incineration or chemical treatment—result in environmental damage and resource depletion. This research introduces a sustainable biotechnological method for the valorization of feather waste utilizing *Gordonia alkanivorans* S7, an actinomycete strain extracted from petroleum plant sludge. This is the inaugural publication illustrating keratinolytic activity in the *Gordonia* genus. The optimization of the degradation process via the Taguchi approach led to the effective biodegradation of untreated home chicken feathers, achieving dry mass loss of up to 99% after 168 h in a mineral medium. The agricultural potential of the obtained keratin hydrolysate, which was high in organic components (C 31.2%, N 8.9%, H 5.1%, and S 1.7%), was assessed. Phytotoxicity tests demonstrated that the feather hydrolysate led to better growth of the indicator plants—*Sorghum saccharatum* and *Lepidium sativum.* The highest values of root growth stimulation were 26% for *S. saccharatum* and 31% for *L. sativum*, at a dose of 0.01%. Shoot growth stimulation was noted only for *L. sativum,* reaching 38% (0.01%), 53% (0.05%), and 37% (0.1%), as compared to the control sample. These results demonstrate the process’s combined economic and environmental benefits, providing a fresh approach to the production of bio-based plant biostimulants and sustainable keratin waste management.

## 1. Introduction

Poultry breeding and processing has experienced numerous transformations over the course of its 8000 year history. The growth of this business has significantly increased over the past 70 years due to advancements in the quality and accessibility of poultry meat. Global chicken production has been steadily increasing on an annual basis. The projected value of the worldwide chicken market is anticipated to reach $429.11 billion by the year 2028. Chicken flesh is essential, as it serves as the primary protein source for individuals in several regions [1].

In the poultry industry, feathers serve as an important byproduct, constituting 5–7% of a chicken’s body weight [2]. Previously, their potential application in both industry and daily life was neglected. They were solely utilized as a filling for cushions and comforters. The unused feathers were either burned or disposed of in landfills, resulting in environmental contamination. The potential value of the feathers was not recognized until the 1990s, when they were processed into meal, which was used as animal feed and in supplements [2,3].

Chicken feathers are composed mainly of keratins, which account for approximately 91% of their structure. Keratins contain disulfide bridges, hydrogen, and covalent bonds, which provide substantial resistance to environmental factors and proteolytic enzymes. Feathers comprise 1% lipids and 8% water. The amino acid composition of feathers mostly consists of cystine, glutamine, proline, and serine, while exhibiting notable shortages in histidine, lysine, tryptophan, glutamic acid, and glycine [4,5].

Feathers are mostly broken down using physicochemical processes, such as through alkaline hydrolysis using substances such as sodium sulphide, sodium hydroxide, or calcium hydroxide, as well as alkaline acid hydrolysis using sodium hydroxide and citric acid. Other methods include heating in a microwave oven, polymerization, and carbonization [6]. However, these technologies are limited by their substantial energy consumption and adverse environmental impacts. Recently, biotechnological methods using intact microbial cells or their enzymes have been employed to degrade keratin. Several methods have been developed to process poultry feathers for reuse, including the production of organic fertilizers, biodiesel, methane, hydrogen, and biodegradable polymers [7,8,9,10,11]. Biologically active peptides derived from keratin residue in feathers, which can be obtained via microbial and enzymatic hydrolysis, are of interest [12,13].

The microbiological processes for processing keratin waste are ecologically sustainable and operate under mild conditions. The microorganisms capable of producing keratinases include bacteria, actinomycetes, and fungi. So far, feathers have been decomposed using strains from the genera *Aeromonas, Amycolatopsis, Bacillus, Brevibacillus, Burkholderia, Chryseobacterium, Fervidobacterium, Geobacillus, Meiothermus, Microbacterium,* and *Paenibacillus. Bacillus* strains are the primary producers of keratinolytic enzymes [14,15,16,17]. The efficacy of keratolytic actinomycetes such as *Streptomyces, Arthrobacter, Actinomadura, Brevibacterium, and Nocardiopsis* [18,19,20,21,22,23,24] and fungi such as *Aspergillus niger, Chrysosporium*, and *Trichophyton ajelloi* has also been confirmed [25,26,27].

The study used the aerobic Gram-positive bacteria *Gordonia alkanivorans* S7 for the biodegradation of chicken feathers. The actinomycete was isolated from effluent originating from petrochemical facilities, as documented by Kwapisz et al. (2008) [28]. The strain has demonstrated its ability to break down difficult-to-biodegrade materials. It has been successfully utilized in the biodegradation of diesel hydrocarbons [29] and in the decomposition of brown coal [30,31].

## 2. Results

### 2.1. Optimization of Household Chicken Feather Degradation

In this study, the impact of five factors on *Gordonia alkanivorans* strain S7′s biodegradation of chicken feathers was evaluated with the use of the Taguchi method. The studied factors included the pH of the culture medium, concentration of the feathers, concentration of the inoculum, temperature, and shaking rate. The Taguchi optimization experiments were conducted according to the design (see the table in Section 4.3, Table 1A,B) and the feather dry mass loss for each trial was determined as the output parameter.

**Figure 1:**
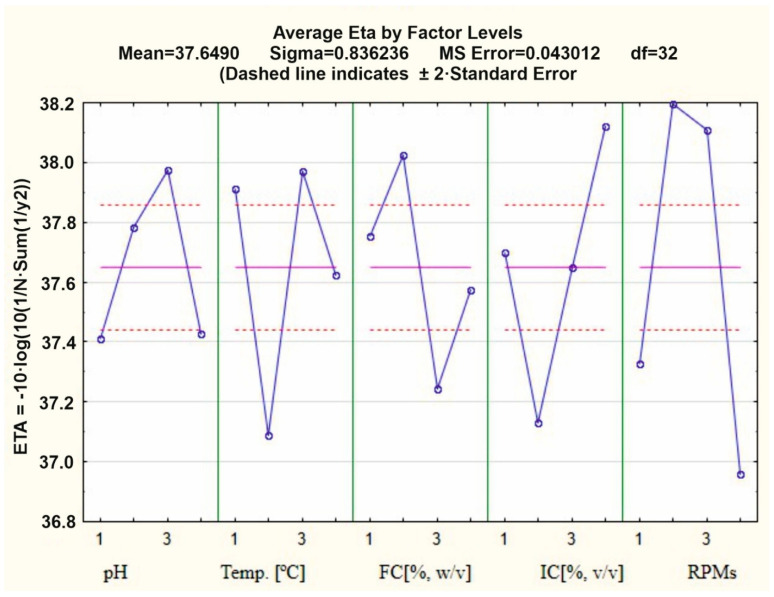
Main effect diagrams for S/N ratios with a larger-the-better objective function of the Taguchi optimized degradation of household feathers using *G. alkanivorans* S7. FC—feather concentration; IC—inoculum concentration.

Based on the contribution ratio of each factor, it can be concluded that the inoculum concentration (47.18%) and shaking speed (46.79%) had the most significant impact on the degradation of chicken feathers by *G. alkanivorans* S7. In contrast, the feather concentration had the least influence on the process (30.35%) among the tested variables. The extent of chicken feather biodegradation ranged from 8.33% ± 3.05 in experiment 9 to 60.43% ± 1.56 in experiment 11. According to the results presented in Figure 1, the most favorable conditions for chicken feather biodegradation were a pH of 7 (factor A at level 3), temperature of 30 °C (factor B at level 3), a feather concentration of 2% *w*/*v* (factor C at level 2), an inoculum concentration of 0.8% (factor D at level 4), and 160 rpm (factor E at level 2). These parameters resulted in the highest observed degradation efficiency.

### 2.2. Biodegradation of Household Chicken Feathers in the Specified Optimal Conditions

The biodegradation of keratin waste was carried out using *G. alkanivorans* S7 over a two week shaking culture of this actinomycete in a mineral medium. The sole source of carbon was chicken feathers, with a concentration of 20 g L^−1^ (dry weight). The experiment was conducted on whole feathers, which if the method is adapted to an industrial use will reduce the expenses related to the biodegradation process by eliminating the cutting or grinding step.

**Figure 2:**
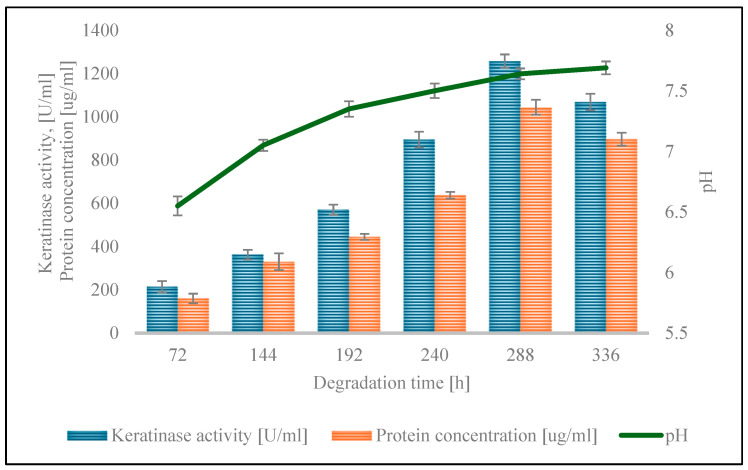
Keratinolytic activity, protein concentration, and pH during the cultivation of *G. alkanivorans* S7 on chicken feathers (*p*-value was 0.05).

The activity of keratinolytic enzymes, protein concentration, and pH changes in the culture medium were assessed during the culture period (Figure 2). The enzyme activity constantly increased throughout the culture, to a peak of 1257.50 ± 30.64 U/mL on the 288th day of the process. A subsequent decline in the value to 1068.33 ± 38.04 U/mL was noted. The maximum protein concentration, derived from extracellular proteins secreted by strain S7 and from feather decomposition, reached 945.76 ± 13.89 µg/mL on the 366th day of culture. The pH of the medium increased from 7.00 ± 0.06 at the beginning of the culture process to 7.69 ± 0.05 after 336 h of culture.

**Figure 3:**
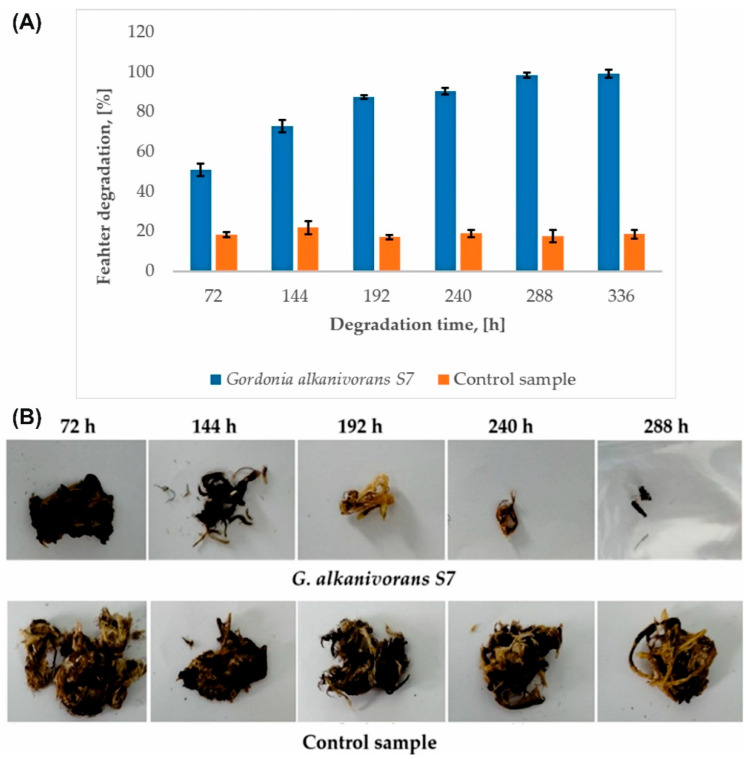
(**A**) Efficiency of chicken feather degradation by *G. alkanivorans* S7 (*p*-value was 0.05). (**B**) Macroscopic view of residues of chicken feathers during biodegradation by *G. alkanivorans* S7.

The *G. alkanivorans* S7 strain is an effective producer of keratinolytic enzymes, which facilitate the intensive decomposition of chicken feathers in a mineral medium (Figure 3A). As a result of the degradation process occurring during the culture, a loss of sample mass was observed. After 72 h of the process, 50.94 ± 3.06% degree of feather biodegradation was obtained, compared to 18.57 ± 1.22% in the control samples. After 288 h, almost complete reduction of the sample was achieved, resulting in mass loss of 98.52 ± 1.23%. Extending the microbiological culture time by an additional 48 h allowed for 100% degradation of the feathers. No solid fragments of the sample were detected in the culture medium. The degree of feather decomposition in all control samples was constant and amounted to 18.90 ± 1.67%. A significant increase in feather degradation was observed in samples inoculated with *G. alkanivorans* S7 compared to the control (*p* < 0.05). A post hoc Tukey’s test confirmed that the degradation significantly increased up to 192 h, after which no statistically significant differences were detected between subsequent time points (*p* > 0.05), indicating that the degradation process had reached saturation. Figure 3B shows images of feather residues after biodegradation next to the control samples.

### 2.3. Morphology of Feathers

**Figure 4:**
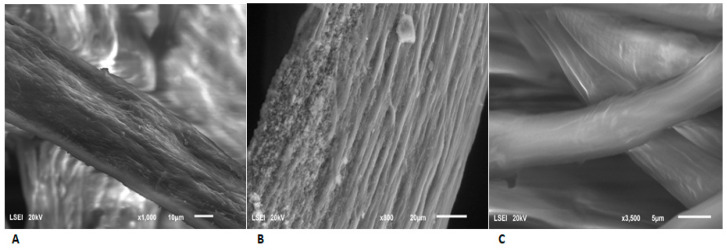
SEM analysis of the feathers: (**A**,**B**) after biodegradation by *G. alkanivorans* S7 for 240 and 288 h, respectively; (**C**) control sample, incubation under experimental conditions for 288 h.

An examination of the microstructural changes in the feathers after biodegradation by *G. alkanivorans* S7 was performed using scanning electron microscopy (SEM). The studies revealed structural changes occurring in the chicken feathers. The control feathers retained their original, compact structure. Nevertheless, the degradation of feathers is readily apparent when they are exposed to microbial activity. Cracking and loss of subsequent layers of the sample were observed. SEM images are presented in Figure 4A–C.

### 2.4. Chemical Structure of Feathers

**Figure 5:**
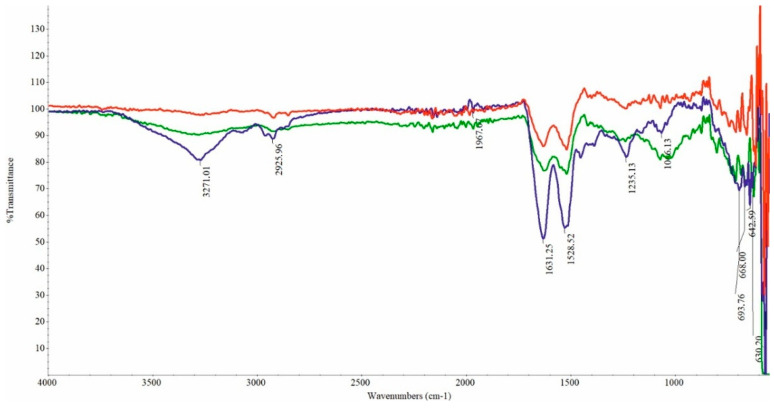
FTIR spectra of chicken feathers before and during biodegradation: blue—“0” sample; red—control sample after 288 h; green—biodegradation sample after 288 h.

FTIR spectra were assessed to analyze the structures of both the undegraded and microbiologically degraded feathers. The characteristic peaks of the examined samples (Figure 5) are comparable and identical to those reported in another study on chicken feather biodegradation [16,32]. The FTIR spectra of all feather samples exhibited typical transmittance associated with amide A, I, II, and III bands. The transmission band region from 3500 cm^−1^ to 3200 cm^−1^ corresponds to the stretching vibrations of -O-H and -N-H, while the range of 3000–2800 cm^−1^ is associated with the symmetric stretching vibrations of -CH_3_, referred to as amide A. The amide I band, observed in the wavenumber range of 1700–1600 cm^−1^, corresponds to the stretching vibrations of carbonyl (CO), while the amide II band, located around 1520 cm^−1^, is indicative of NH bending and CH stretching vibrations. The band attributed to amide III, spanning 1220 cm^−1^ to 1300 cm^−1^, is associated with the CN stretching and CO bending ratios. A band matching to S-O was found in the sulfoxide region at 1071 cm^−1^. The band was of low intensity in control feathers, whereas the degraded feathers displayed high intensity.

### 2.5. Effect of Inoculum Concentration and Feather Type on the Rate of Their Biodegradation

**Figure 6:**
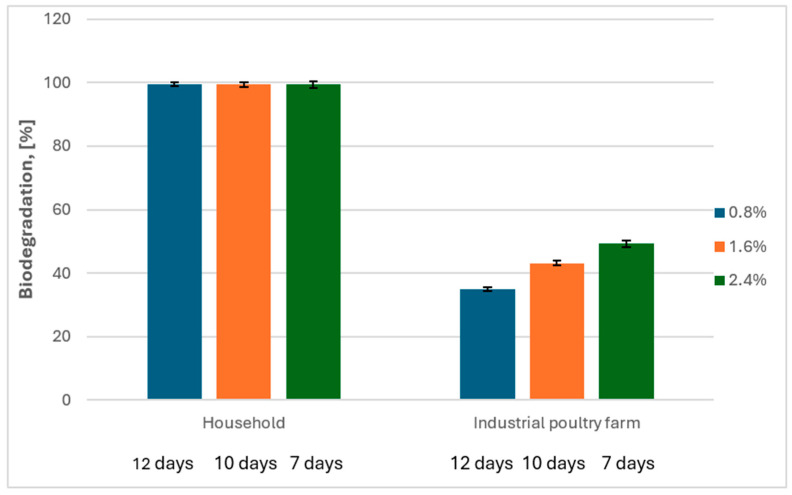
The effect of the inoculum concentration and feather type on the rate of their biodegradation by *G. alkanivorans* S7 (*p*-value was 0.05).

Based on the main effect diagrams for S/N ratios with a larger-the-better objective function of the Taguchi optimization (Figure 1), it was observed that the inoculum concentration is one of the most important parameters influencing the feather degradation process by the G. *alkanivorans* S7 strain. Therefore, an experiment was conducted to determine the effect of higher inoculum concentrations of 0.8, 1.6, and 2.4% (*v*/*v*) and the type of feather on the biodegradation rate. Two types of feathers, i.e., from a household and an industrial poultry farm, were subjected to decomposition, which are characterized in Section 4.2. This was done also to ascertain whether an increase in inoculum concentration might reduce the time required for complete biodegradation of feathers. In the case of biodegradation of feathers from a household, increasing the inoculum concentration reduced the time required to achieve 100% feather mass loss. For the concentration of 2.4% (*v*/*v*), this time was 7 days. The experiment conducted on the second type of substrate confirmed that feathers from poultry farms are poorly degraded by *G. alkanivorans* S7. The use of inoculum at concentrations of 0.8, 1.6, and 2.4 led to feather mass losses of 35, 43, and 48%, respectively (Figure 6). Tukey’s post hoc test showed no statistically significant differences in biodegradation between the 7, 10, and 12 day treatments under household conditions (*p* > 0.05), suggesting that the process could be effectively completed within 7 days. In contrast, under industrial poultry farm conditions, although a slight increase in biodegradation was observed with shorter processing times, the differences were not statistically significant (*p* > 0.05).

### 2.6. Chicken Feather Hydrolysate as a Potential Fertilizer

**Figure 7:**
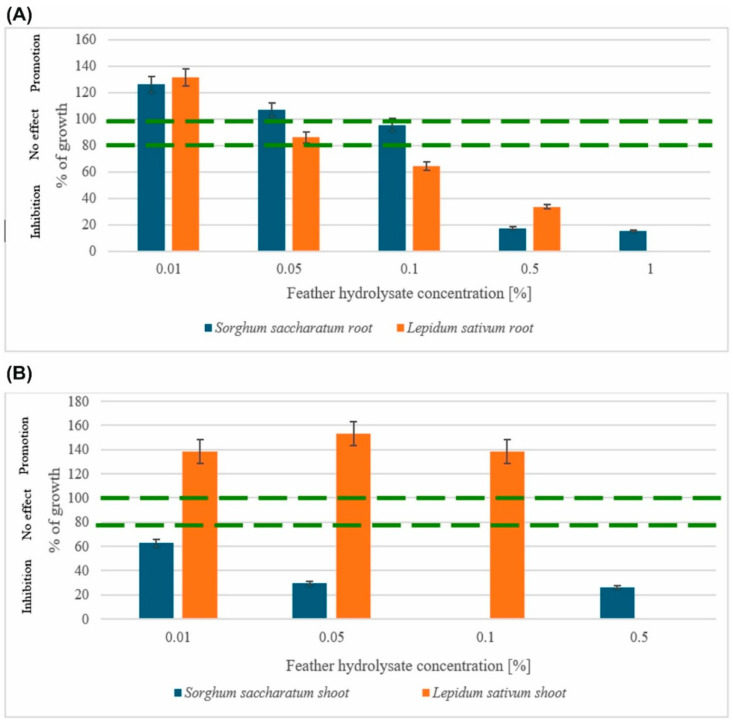
Impact on growth-promoting ability of used plants: (**A**) roots and **(B**) shoots of the chicken feather hydrolysate (*p*-value was 0.05).

To evaluate the feasibility of using the residues from the feather biodegradation process carried out by the *G. alkanivorans* S7 strain, an elemental analysis of the hydrolysate was conducted (Section 4.7). The results of the elemental analysis of the feather hydrolysate were as follows: 8.940 ± 0.023% nitrogen; 31.156 ± 0.094% carbon; 5.077 ± 0.038% hydrogen; 1.65 ± 0.042% sulfur. The protein concentration in the sample was 896.11 ± 10.62 µg/mL. This composition makes the resulting product suitable for potential application in agriculture as a fertilizer. For confirmation, phytotoxicity tests were performed using two indicator plants—*Sorghum saccharatum* and *Lepidium sativum* (cress). The germination of the studied plants, irrigated with varying concentrations of feather hydrolysate solution (0.001%, 0.05%, 0.5%, 1%), was evaluated and compared to the results of the control samples irrigated with water. The obtained results are presented in Figure 7.

A significant increase in the growth rate of the plant roots was observed in experiments using low doses of chicken feather hydrolysate of 0.01% and 0.05%. The maximum root growth stimulation values were 26% for *S. saccharatum* and 31% for *L. sativum*, at a dose of 0.01%. The stimulation of shoot growth was noted only for *L. sativum*, reaching 38% (dose 0.01%), 53% (dose 0.05%), and 37% (dose 0.1%), as compared to the control sample. Higher hydrolysate concentrations of 0.5% and 1% resulted in the inhibition of plant growth.

## 3. Discussion

Actinobacteria are a group of saprophytic, Gram-positive bacteria found in both terrestrial and aquatic ecosystems. Morphologically, they resemble fungi and are uniquely characterized by the formation of branching hyphae, which can develop into mycelia. These microorganisms possess a broad enzymatic repertoire, enabling them to use a wide range of substrates as nutrient sources [33].

The extensive enzymatic apparatus of actinomycetes includes members that produce keratinolytic enzymes. Examples of actinobacteria-producing keratinases are those of the genera *Streptomyces*, *Arthrobacter*, *Actinomadura*, *Brevibacterium*, and *Nocardiopsis*. Most keratinases originating from the genus *Streptomyces* have been described, such as the keratinolytic serine proteinase from *S. pactum* DSM 40530 [34] and the keratinases from *S. gulbargensis* [35], *S. albidoflavus* [18], *S. thermoviolaceus* SD8 [36], *S. aureofaciens* K13 [20], *Streptomyces* sp. 2M21 [37], and *S. swerraensis* KN23 [38]. Other examples include keratinases from *Arthrobacter* sp. NFH5 [21], *Actinomadura keratinilytica* strain Cpt20 [39], *Brevibacterium luteolum* MTCC 5982 [22], *Nocardiopsis* sp. TOA-1 [40], and *Nocardiopsis* sp. SD5 [41]. It should be emphasized that despite the development of knowledge about the keratinolytic abilities of actinobacteria, the best producers of these enzymes, demonstrating the highest efficiency in the biodegradation of keratin waste, remain bacteria belonging to the genus *Bacillus*.

Actinobacteria include aerobic, non-motile, non-sporulating, and catalase-positive bacteria of the genus *Gordonia*, belonging to the order *Mycobacteriales* [42]. This genus comprises 55 validly published species [43]. The colony morphology of *Gordonia* species varies considerably, from slimy, smooth, and glossy forms to irregular and rough textures; differences may also occur within a single species, depending on the medium used for growth. *Gordonia* is well characterized for its capacity to metabolize structurally diverse and often recalcitrant compounds, including those present in fossil fuels, both substituted and non-substituted hydrocarbons, widespread toxic environmental pollutants, various xenobiotics, and natural compounds that are not readily biodegradable. These characteristics render these bacteria interesting for scientific research and valuable in biotechnology [42,44].

This study used the strain *Gordonia alkanivorans* S7, isolated from petroleum plant sludge. Numerous properties of this strain have been evaluated. It is an efficient aerobic denitrifier, which may enhance the biodegradation of diesel oil hydrocarbons [28,45]. The respiratory nitrate reductase (*Nar*) from this strain was isolated, purified, and partially characterized [29]. *G. alkanivorans* S7 has been proven to biosolubilize brown coal [30,31]. To date, no strain from the genus *Gordonia* has been reported to possess keratinolytic activity. Our study is the first to demonstrate this capability in *G. alkanivorans* S7, thereby expanding the known metabolic profile of the genus beyond hydrocarbon and xenobiotic degradation.

Our preliminary studies on the biodegradation of chicken feathers using *G. alkanivorans* S7 (unpublished) concentrated on several poultry feathers types, differing in origin, particularly from industrial poultry farms and households. Poultry farm feathers were determined to be more difficult for *G. alkanivorans* S7 to biodegrade. The initial chemical sanitation of feathers at the poultry farm likely adversely impacts the growth and activity of the used microorganism. Therefore, this study focused on the biodegradation of household feathers.

The Taguchi method was employed to optimize the conditions for chicken feather biodegradation by *G. alkanivorans* S7, evaluating five key factors. This statistical optimization technique was selected due to its effectiveness in studying complex multivariable systems with a reduced number of experimental trials compared to full factorial designs. The Taguchi method employs orthogonal arrays to investigate the influence of multiple parameters and their interactions systematically, making it particularly suitable for microbial biodegradation processes that involve numerous environmental and operational variables. The selected factors—the initial pH of the culture medium, feather concentration (*w*/*v*), inoculum concentration (*v*/*v*), incubation temperature, and shaking speed (rpm)—were chosen based on preliminary tests and the existing literature. Each factor was tested at four levels according to the L16 orthogonal array design (see the table in Section 4.3, Table 1A,B). The biodegradation efficiency was assessed by measuring the percentage loss of feather dry mass in each experimental run, which served as the response variable. A data analysis was conducted using the signal-to-noise (S/N) ratio with a “larger-the-better” objective function, aiming to maximize feather degradation. This approach not only identified the most effective combination of operational conditions but also revealed the relative contribution of each factor to the process performance. The results derived from the optimization of domestic chicken feather degradation via the Taguchi technique reveal that the inoculum concentration significantly influences the degradation efficiency by *G. alkanivorans* S7. Further biodegradation studies were performed utilizing elevated inoculum concentrations of 1.6% and 2.4% (*v*/*v*). Two categories of feathers were exposed to decomposition—those from a domestic poultry farm and those from an industrial poultry farm. An inoculum concentration of 2.4% facilitated a mass loss of feathers from the household amounting to 99.38 ± 0.53% within 7 days. For the latter category of feathers, a notably reduced result of 49.32 ± 1.47% was recorded after 7 days but the inoculum concentration exhibited no significant impact on the decomposition rate. Given the differences in feather dimensions, with the length and width of the feathers from the chicken farm being 2 and 3.5 times smaller than those from the household, it can be assumed that smaller feathers may degrade more readily due to microbial activity. However, the data indicate that the breakdown of feathers from the chicken farm was significantly less successful, perhaps due to the pre-treatment of these feathers in the poultry facility.

The biodegradation process, with the parameters optimized using the Taguchi method, was conducted during the cultivation of *G. alkanivorans* S7 in a mineral medium (pH 7.0), using chicken feathers (20 g L^−1^ dw) as the sole carbon source. Notably, from an industrial application perspective, the feathers remained uncrushed. They were introduced whole into the culture medium, which was inoculated with a pre-culture of 0.8% *v*/*v*. The keratin material degradation process was carried out for 336 h at a temperature of 30 °C, with culture shaking at 160 rpm. During the cultivation of the strain, changes in the pH of the medium, the concentration of extracellular protein, the activity of keratinolytic enzymes, and most importantly the loss of dry feather mass were examined. The degradation of feathers by *G. alkanivorans* S7 in the mineral medium demonstrated that it could utilize feathers as the only source of nutrients. For the household feathers, the pH of the culture medium gradually increased from 7.00 ± 0.03 to 7.69 ± 0.05 at 336 h of cultivation. It should be emphasized that the increase in pH of the culture medium is an important indicator of the ongoing hydrolysis of keratin [46]. During the process of keratin degradation, oxygen deamination occurs, which ultimately leads to the formation of ammonia, which increases the pH of the medium. Similar pH increases during feather degradation were previously reported by Riffel et al. (2003) [46] and Yadav and Khosla (2021) [47].

The concentration of soluble protein gradually increased during the cultivation, reaching a maximum of 945.76 ± 13.83 µg/mL after 366 h of the process. The activity of the keratinase reached a maximum after 288 h of cultivation, i.e., 1257.50 ± 30.64 U/mL. Extending the cultivation for an additional 48 h resulted in a decrease in the activity of the specified enzyme, possibly related to cell autolysis. The apparent inactivation of the enzyme may also be due to the accumulation of metabolic end products in the medium or depletion of nutrients in the medium [47]. After 288 h of strain cultivation, 98.52 ± 1.23% of the feather dry mass is lost. Again, extending the cultivation time by an additional 48 h facilitated complete degradation of the feathers. No discernible solid feather particles were detected in the post-culture liquid. The loss of feather dry mass in the control samples remained constant during the process (18.90 ± 1.67%). This loss may be due to heat treatment of the feathers (sterilization), which was performed before culturing the strain, as well as mechanical degradation (shaking during culturing). Comparing the keratinase activity between strains is inherently challenging due to the lack of a standardized assay method and the use of a variety of substrates. Various materials have been used in activity studies, such as chicken feathers, keratin, keratin azure, azokeratin, azocasein, and others [15]. Each substrate has a different effect on the measured enzyme activity. These methodological inconsistencies complicate direct comparisons of keratinase activity between strains. The research by Nnolim and Nwodo [48] indicates that the *Bacillus* sp. CSK2 strain reached a peak keratinase activity rate of 1539.09 ± 68.14 U/mL after 48 h of degradation process under optimized conditions, using a medium containing chicken feathers (7.5 g L^−1^) and maltose (2.0 g L^−1^), initial pH of 5.0, incubation temperature 30 °C, and agitation speed 200 rpm. In the described studies, azure keratin was used to determine the keratinolytic activity. The same substrate and very similar test conditions were used to analyze the activity of keratinases from *G. alkanivorans* S7; hence, the activities of these two strains can be compared. The strain S7 reached a maximum keratinase activity of 1257.50 U/mL after 288 h of cultivation in a mineral medium containing a higher concentration of chicken feathers (20 g L^−1^) at 30 °C, with shaking at 160 rpm and without additional supplementation. Although this activity was marginally reduced and necessitated an extended incubation period, it is noteworthy that this level of enzyme production was attained without the incorporation of readily metabolizable carbon sources, underscoring the strain’s potential for biotechnological applications in the cost-effective valorization of feather waste.

The majority of published studies on the keratinolytic capabilities of actinomycetes have concentrated on the isolation, purification, and characterization of keratinases, testing only the purified proteins for keratin biodegradation [18,21,35,36]. In contrast, an excellent example of the use of an actinomycete for feather degradation is *Streptomyces* sp. SCUT-3, where the degradation of this material was evaluated throughout the strain culture. It could completely degrade both the plumage and shafts of white chicken feathers (uncrushed) within 36 h in mineral medium with 1% chicken feather supplement, resulting in a feather weight loss of 94%. It was also found that the SCUT-3 strain was able to completely degrade feathers within 8 days at their concentration of 10% in the culture medium, while at a concentration of 40%, a degradation level of 57% was noted on day 6 [24]. In subsequent studies, the SCUT-3 strain with co-overexpression of much higher-efficiency keratinases was developed and used for solid-state feather fermentation. The keratinase activity of the recombinant strain was 16 times more than that of the wild type, and the feather degradation was performed for only 2.5 days at a feather-to-water ratio of 1:1.5 [49]. The time required for the degradation of uncrushed household feathers to 99% (with a feather concentration of 2% *w*/*v*) utilizing the *G. alkanivorans* S7 strain is extended to 7 days. It should be emphasized that these are preliminary studies, and future research studies will be primarily concentrated on reducing the process’s duration. The SCOUT-3 strain-related investigations [24,49] did not include any information regarding the inoculation of the feather substrate, which has the potential to significantly influence the rate of feather decomposition. Another example of microorganism from *Streptomyces* genera is *S. exfoliatus* CFS 1068, which caused a weight loss of 83% in feather weight in 6 days at 28 °C in shake cultures (150 rpm). Nevertheless, the decomposition of the feathers was likely accelerated by the use of feather meal (1% *w*/*v*) rather than whole feathers in this particular case [50].

The most efficient keratinase producers are strains of the *Bacillus* species. Abdelmoteleb et al. (2023) [51] investigated two strains, *S. netropsis* A-ICA and *B. subtilis* ALICA, for their production of keratinases and their ability to biodegrade feathers. The study used chicken feathers collected from a chicken slaughter market, which were pre-cleaned, dried, and cut into 2–3-cm-long pieces. The highest keratinase activity for *S. netropsis* A-ICA and *B. subtilis* ALICA (114 and 136 U/mL) was obtained on the 5th and 3rd day of incubation using 1% (*w*/*v*) chicken feathers in the culture medium. The greatest substrate weight loss was 84% for *S. netropsis* A-ICA at day 7 and 86% for *B. subtilis* ALICA at day 5. The research by Yadav and Khosla (2021) [47] showed that the *B. cereus* H16 strain effectively degraded 94% of the feathers (fragmented into approximately 1 cm pieces), yielding 9.70 mg/mL of protein, inclusive of the keratinase activity (196 U/mL) during a span of 3 days. The strain was cultured on a mineral medium containing feathers at 1% *w*/*v*. Maintaining the same process parameters, the *B. cereus* H3(2) strain showed keratinolytic activity of 165 U/mL, and the protein concentration in the post-culture liquid was 7.78 mg/mL, while degrading 79.7% of the feathers [47].

We evaluated the effectiveness of the feather degradation by *G. alkanivorans* S7 under conditions optimized specifically for this strain using the Taguchi method. However, these conditions differ from those commonly used in studies involving species particularly efficient in degrading keratin such as *Bacillus* or *Streptomyces*. Consequently, it must be acknowledged that these comparisons are limited by the significant diversity in the experimental designs and the substrates employed (extent of feather fragmentation).

Although the keratinolytic abilities of certain actinobacteria, such as *Streptomyces*, *Arthrobacter*, and *Nocardiopsis*, are well documented, the genus *Gordonia* has not been previously reported to possess keratin-degrading capabilities. The discovery of keratinolytic activity in *G. alkanivorans* S7 is, therefore, novel and significantly expands the known metabolic repertoire of this genus. This work highlights feather biodegradation through entire cells, in contrast to several studies that have concentrated on characterizing pure keratinases. We used unprocessed whole chicken feathers and assessed the keratin degradation in an unsupplemented mineral medium, providing a more application-relevant model. Despite the degradation efficiency and rate in our system being frequently lower or comparable to those attained in optimized systems utilizing *Bacillus* strains or recombinant *Streptomyces*, the keratinolytic activity exhibited by *G. alkanivorans* S7 underscores its potential as a viable candidate for sustainable and industrially significant feather waste valorization.

Following the degradation results, we subsequently assessed the agricultural application potential of the resulting hydrolysate. The feather hydrolysate obtained using *G. alkanivorans* S7 was found to contain averages of 31.2% C, 8.9% N, 5.1% H, and 1.7% S, as determined by the elemental analysis. Consequently, the biofertilizer potential of the product was evaluated and its suitability for plant biostimulation was assessed. This was accomplished by conducting phytotoxicity assays (Phytotoxkit^®^ 3 day test), which were carried out on two indicator plants, *Sorghum saccharatum* and *Lepidium sativum,* using hydrolysate in concentrations from 0.01% to 1% (*w*/*v*). The root growth stimulation rate was 26% for *S. saccharatum* and 31% for *L. sativum*, at the lowest dose of 0.01%. The use of higher concentrations inhibits the growth of the roots. Shoot growth stimulation was noted only for *L. sativum*, reaching 38% (0.01% dose), 53% (0.05% dose), and 37% (0.1% dose). These preliminary findings suggest that the hydrolysate that was obtained may be employed in agriculture to support plant growth. Comparable findings have been documented by other researchers. Numerous studies have confirmed that the use of feather hydrolysate stimulates seed germination and plant growth in pot and field conditions [24,52,53]. This hydrolysate contains peptides, soluble proteins, and amino acids that support the growth of microorganisms in the rhizosphere, which promotes the uptake and use of nutrients from the soil. Increases in water capacity, soil mineral content, and C/N ratio have also been reported [53,54]. Experiments conducted with *Streptomyces* sp. SCUT-3 fermented feathers confirmed that it is a good plant amino acid liquid fertilizer [24]. Feather hydrolysate was added to rice grown in soil (50 mg of amino acids from 10% fermented broth in 50 g soil) and in hydroponics (50 mg L^−1^ of amino acids). Both methods showed growth stimulation of rice. In the study by Kaur et al. (2021) [55], the stimulating potential of chicken feather hydrolysate produced by *B. aerius* NSMk2 was confirmed on the growth of mung bean (*Vigna radiata*) in pot conditions. Extending the research on the effect of the tested feather hydrolysate on a wider range of plant species, conducting field trials, and assessing the impact on soil microbial communities are the next necessary steps to confirm its application as a plant-growth-stimulating biofertilizer.

The confirmed keratinolytic activity of the *G. alkanivorans* S7 strain represents a novel finding within this genus. These findings broaden the known functional spectrum of *Gordonia* species, which have primarily been examined for their ability to degrade hydrocarbons and their metabolic versatility toward xenobiotic compounds. It should also be emphasized that these are preliminary results. Future research studies will focus on process intensification and strain improvement for enhanced efficiency and reduced degradation times. Future studies will also investigate the underlying molecular mechanisms, identifying specific enzymatic activities such as sulfite reductases that may be involved in the reduction of disulfide bonds during keratin degradation by this strain and assessing the scalability of the process for potential industrial applications.

## 4. Materials and Methods

### 4.1. Microorganism

The strain of *Gordonia alkanivorans* S7 originated from the resources of the Institute of Molecular and Industrial Biotechnology, Lodz University of Technology. The strain was stored at 4 °C on Luria–Bertani (LB) agar.

### 4.2. Chemicals and Materials

All chemicals used in this study were of analytical grade. The keratin azure was purchased from Sigma-Aldrich (St. Louis, MO, USA). Two types of chicken feathers were analyzed: (1) from the household (Lipce Reymontowskie, Poland), for which the feather rachis length and width were 125 ± 31 and 3.9 ± 0.4 mm, respectively, and the vane color was white-brown; (2) from the largest poultry producer in Poland, for which the feather rachis length and width were 64 ± 10 and 1.1 ± 0.2 mm, respectively, and the vane color was snow-white (these feathers were subjected to a sanitation process at the manufacturer’s headquarters). Both types of feathers were additionally washed, degreased with detergent, thoroughly rinsed with distilled water, and autoclaved (121 °C, 100 kPa, 15 min). Wet feathers were used for the biodegradation tests. In order to determine the dry weight of the feathers, they were dried in an oven at a temperature of 105 °C to a constant weight.

### 4.3. Optimization of Household Feather Biodegradation Using Taguchi Methodology

The biodegradation of household feathers was optimized using the Taguchi methodology, which employs orthogonal arrays to examine multiple variables with minimal experimental effort. The orthogonal array L’16 consisted of the maximum number of input parameters, i.e., 5 parameters at 4 levels (Table 1A,B). The factors studied included the temperature (20–35 °C), pH (5–8), feather concentration (1–4% *w*/*v*), inoculum concentration (0.2–0.8% *v*/*v*; OD_550_ 0.954 ± 0.031), and shaker speed (140–200 rpm). The biodegradation of the feathers was carried out in 500 mL Erlenmeyer flasks containing 50 mL of mineral medium ([g L^−1^]: MgSO_4_ 0.2; CaCl_2_ 0.2, KH_2_PO_4_ 10, K_2_HPO_4_ 10, NH_4_NO_3_ 10, FeCl_3_ 0.05) with the addition of an appropriate amount of feathers. The feather decomposition time was 336 h. The optimization of the degradation conditions was evaluated based on the degree of feather degradation.

**Table 1:** Experimental conditions for the Taguchi design.

**(** **A** **)**						
**Symbol**	**Factor**		**Level**			
			**L1**	**L2**	**L3**	**L4**
**A**	pH		5	6	7	8
**B**	Temperature (°C)		20	25	30	35
**C**	Feather concentration (% *w*/*v*)		1	2	3	4
**D**	Inoculum (% *v*/*v*)		0.2	0.4	0.6	0.8
**E**	RPM		140	160	180	200
**(B)**						
**Factor**	**pH**	**Temperature (°C)**	**Feather Concentration** **(% *w*/*v*)**		**Inoculum (%)**	**RPM**
**Run**						
**1**	L3	L1	L3		L4	L2
**2**	L1	L2	L2		L2	L2
**3**	L2	L4	L3		L2	L1
**4**	L4	L4	L1		L3	L2
**5**	L2	L1	L2		L3	L4
**6**	L1	L4	L4		L4	L4
**7**	L2	L2	L1		L4	L3
**8**	L4	L3	L2		L4	L1
**9**	L3	L4	L2		L1	L3
**10**	L2	L3	L4		L1	L2
**11**	L4	L2	L3		L1	L4
**12**	L4	L1	L4		L2	L3
**13**	L1	L1	L1		L1	L1
**14**	L1	L3	L3		L3	L3
**15**	L3	L2	L4		L3	L1
**16**	L3	L3	L1		L2	L4

Indices 1, 2, 3, and 4 represent the levels of the factors, with the symbol L denoting level. The objective of the optimization was to determine the input factor levels that would maximize the degradation of chicken feathers. The system’s response was characterized by a degree of feather degradation. In order to reduce systematic errors, each configuration was subjected to three experimental repetitions. The parameters with the highest desired value were selected, and the signal-to-noise (S/N) ratio, indicating controllable factors in relation to confounding factors, was calculated using the following formula:

(1) $$\left(S/N\right)HB=-10log\left[\left(\frac{1}{n}\right)\sum \left(\frac{1}{yi^{2}}\right)\right]$$

Let *i* represent the total number of measurements, *n* denote the number of measurements for a specific feature, and *y* indicate the measured feature.

### 4.4. Feather Biodegradation Tests in the Specified Optimal Conditions

The biodegradation of the feathers was carried out in 500 mL Erlenmeyer flasks containing 50 mL of mineral medium ([g L^−1^]: MgSO_4_ 0.2; CaCl_2_ 0.2, KH_2_PO_4_ 10, K_2_HPO_4_ 10, NH_4_NO_3_ 10, FeCl_3_ 0.05). The medium was supplemented with feathers in the amount of 20 g L^−1^ (dw), which were not subjected to any mechanical pretreatment. The pH was adjusted to 7.00 ± 0.06 with 1M NaOH. The medium containing feathers was sterilized (121 °C, 100 kPa, 20 min) and subsequently inoculated with *G. alkanivorans* S7 at a concentration of 0.8% (*v*/*v*), OD_550_ 0.954 ± 0.031. The inoculum was obtained by culturing *G. alkanivorans* S7 on LB medium (Luria Broth; [g L^−1^]: casein peptone 10, yeast extract 5, NaCl 10) at 30 °C, on a rotary shaker at 160 rpm for 12 h. The biodegradation of feathers was conducted for a maximum duration of 336 h at 30 °C, with the flasks agitated at 160 rpm. Samples were collected for our analysis after two or three days. The decreases in feather dry weight, keratinase activity, and protein concentration were assessed in the samples.

### 4.5. Determination of the Degree of Feather Degradation

After the culture of *G. alkanivorans* S7, the feathers remaining in the medium were separated by filtration (Schott G3 filter funnel, Duran Group, Mainz, Germany). To remove the soluble materials and bacterial cells, the remaining feathers fragments were thoroughly washed with distilled water (3 times) and ethanol (3 times) and subsequently dried in an oven at 105 °C to a constant weight. The degradation of the feathers was expressed as a percentage loss of feather mass relative to their initial dry mass. The experimental control was a sample of feathers not inoculated with *G. alkanivorans* S7.

(2) $$Degree\;of\;feather\;degradation\;\left[\%\right]=\frac{Initial\;weight\;\left(d.w.\right)-Final\;weight(d.w.)}{Initial\;weight\;(d.w.)}\times 100\%$$

### 4.6. Determination of Keratinase Activity and Protein Concentration

The keratinolytic activity was determined using keratin azure as a substrate, following a modified protocol described by Nnolim et al. (2020) [48]. First, 0.5 mL of crude enzyme solution was added to 0.5 mL of 10 g/L of keratin azure in 0.1 M of Tris HCl buffer (pH 8.0). The samples were incubated at 50 °C for 1 h at 200 rpm. The mixture was placed in ice cold water for 10 min to stop the reaction, followed by centrifugation at 10,000 rpm for 20 min to remove the unreacted substrate. The supernatant was spectrophotometrically measured for the release of the azo dye at OD = 595 nm. Two control samples were prepared in a similar way to the actual samples. In the first control, the enzyme was replaced by 0.1 M of Tris HCl buffer. In the second one, no substrate (keratin azure) was introduced. One unit (U) of keratinase activity was defined as the amount of enzyme causing an increase of 0.01 absorbance at OD = 595 nm in one hour under given conditions.

The protein concentration was determined according to the Lowry method [56] using bovine serum albumin (Sigma-Aldrich, USA) as a protein standard.

### 4.7. Elemental Analysis

The percentages of nitrogen, carbon, hydrogen, and sulfur in sterilized (121 °C, 100 kPa, 20 min) and lyophilized feather hydrolysates (freeze-dried at −30 °C, 0.37 mbar, using an Alpha 1–4 freeze dryer (Christ, Osterode am Harz, Germany)) were determined with an Elemental Vario El Cube CHNS analyzer (Elementar, Langenselbold, Germany). The fundamental principle of the quantitative CHNS analysis involves the high-temperature oxidative burning of materials. The gaseous combustion products are purified and segregated in absorption columns for specific components (nitrogen, carbon dioxide, sulfur dioxide, water vapor) and subsequently detected in the measurement cell of the thermal conductivity detector (TCD).

### 4.8. Fourier Transform Infrared Spectroscopy (FTIR)

The characteristic functional groups of chicken feathers, both treated with *G. alkanivorans* S7 and the initial feathers, were identified using Fourier transform infrared spectroscopy (FTIR) with a Thermo Scientific NICOLET 6700 FTIR Spectrophotometer (Waltham, MA, USA). The spectra were analyzed in transmittance mode in the wavelength range of 4000–400 cm^−1^ at room temperature.

### 4.9. Scanning Electron Microscopy Analysis of Feathers

Images of the SCUT-3 feathers after various days of biodegradation were obtained using an ultra-high-resolution scanning electron microscope (SEM), (Carl Zeiss, Oberkochen, Germany).

### 4.10. Plant Growth Promotion Tests

The hydrolysate obtained via the degradation of chicken feathers under optimal conditions (point 2.4., time 336 h) was sterilized (121 °C, 100 kPa, 20 min) and then freeze-dried (−30 °C, 0.37 mbar, Alpha 1–4 freeze dryer, Christ, Germany). The effectiveness of these hydrolysates in enhancing plant growth was evaluated in soil using the Phytotoxkit^®^ test (MicroBioTests Inc., Gent, Belgium). In bioassays, the inhibition, presence, and enhancement of seed germination were meticulously observed after a period of three days. The Phytotoxkit assays were conducted using the standard protocol of this test [57] utilizing seeds from *Sorghum saccharatum* and *Lepidium sativum*. The concentrations of chicken feather hydrolysates ranged from 0.01% to 1% (*w*/*v*).

### 4.11. Statistical Analysis

Statistica 14.0 software was utilized for calculating the mean values and standard deviations and conducting a single-factor ANOVA. The analyses were conducted in triplicate. Tukey’s test was employed to assess the differences between individual means and the control mean ± standard deviation. Significance was established at *p* = 0.05, with *p*-values ≤ 0.05 being significant.

## 5. Conclusions

This study is the first to demonstrate the potential of the *Gordonia alkanivorans* S7 actinomycete for the biodegradation of poultry feathers. The use of the examined strain can facilitate the creation of a straightforward and eco-friendly technique for recycling feather waste, while also highlighting aspects that impede microbiological biodegradation in feather processing. The results showed that *G. alkanivorans* S7 is highly effective in feather biodegradation. After 168 h of cultivation in a mineral medium using household chicken feathers as the sole carbon source, 99% of the feather dry mass was degraded, underscoring the strain’s strong keratinolytic potential. Additionally, research has demonstrated that the hydrolysate produced as a result of feather degradation is a valuable source of proteins (including keratinolytic enzymes) that can be employed in various industries, such as in agriculture as fertilizers. The chemical analysis revealed that the hydrolysate is rich in nutrients, containing 31.2% carbon, 8.9% nitrogen, 5.1% hydrogen, and 1.7% sulfur, confirming its potential for agricultural use. The phytotoxicity tests further supported this by showing enhanced growth of the indicator plants *Sorghum saccharatum* and *Lepidium sativum*. The highest levels of root growth stimulation were observed at a concentration of 0.01%, with increases of 26% for *S. saccharatum* and 31% for *L. sativum*. The shoot growth stimulation rates in *L. sativum* reached 8%, 53%, and 37% at concentrations of 0.01%, 0.05%, and 0.1%, respectively, as compared to the control. The developed method can contribute to reductions in environmental pollution associated with keratin waste management and facilitate the creation of a new, industrially useful product.

## Data Availability

Data are contained within the article.
